# Evaluation of Pulmonary Nodules by Radiologists vs. Radiomics in Stand-Alone and Complementary CT and MRI

**DOI:** 10.3390/diagnostics14050483

**Published:** 2024-02-23

**Authors:** Eric Tietz, Gustav Müller-Franzes, Markus Zimmermann, Christiane Katharina Kuhl, Sebastian Keil, Sven Nebelung, Daniel Truhn

**Affiliations:** 1 Department of Diagnostic and Interventional Radiology, RWTH Aachen University Hospital, Pauwelsstr. 30, 52072 Aachen, Germany; gumueller@ukaachen.de (G.M.-F.); ckuhl@ukaachen.de (C.K.K.); snebelung@ukaachen.de (S.N.); dtruhn@ukaachen.de (D.T.); 2 Department of Diagnostic and Interventional Radiology, Medical Faculty, University Dusseldorf, Moorenstr. 5, 40225 Dusseldorf, Germany

**Keywords:** CT, MRI, pulmonary nodule, artificial intelligence, radiomics

## Abstract

Increased attention has been given to MRI in radiation-free screening for malignant nodules in recent years. Our objective was to compare the performance of human readers and radiomic feature analysis based on stand-alone and complementary CT and MRI imaging in classifying pulmonary nodules. This single-center study comprises patients with CT findings of pulmonary nodules who underwent additional lung MRI and whose nodules were classified as benign/malignant by resection. For radiomic features analysis, 2D segmentation was performed for each lung nodule on axial CT, T2-weighted (T2w), and diffusion (DWI) images. The 105 extracted features were reduced by iterative backward selection. The performance of radiomics and human readers was compared by calculating accuracy with Clopper–Pearson confidence intervals. Fifty patients (mean age 63 +/− 10 years) with 66 pulmonary nodules (40 malignant) were evaluated. ACC values for radiomic features analysis vs. radiologists based on CT alone (0.68; 95%CI: 0.56, 0.79 vs. 0.59; 95%CI: 0.46, 0.71), T2w alone (0.65; 95%CI: 0.52, 0.77 vs. 0.68; 95%CI: 0.54, 0.78), DWI alone (0.61; 95%CI:0.48, 0.72 vs. 0.73; 95%CI: 0.60, 0.83), combined T2w/DWI (0.73; 95%CI: 0.60, 0.83 vs. 0.70; 95%CI: 0.57, 0.80), and combined CT/T2w/DWI (0.83; 95%CI: 0.72, 0.91 vs. 0.64; 95%CI: 0.51, 0.75) were calculated. This study is the first to show that by combining quantitative image information from CT, T2w, and DWI datasets, pulmonary nodule assessment through radiomics analysis is superior to using one modality alone, even exceeding human readers’ performance.

## 1. Introduction

Pulmonary nodules are defined as well- or poorly defined radiographic opacities with a diameter ≤ 3 cm, surrounded by lung tissue [1,2]. Incidental pulmonary nodules are frequently found on routinely performed computed tomography (CT) scans of the chest. In the United States, they are detected in approximately 30% of chest CT [3]. In addition, lung cancer screening programs using low-dose chest CT (LDCT) have become more widespread, with detection rates as high as 51% [4,5]. Most studies report that less than 10% of nodules are malignant [6]. The challenge is to distinguish between the more common benign nodules that do not require follow-up and the much rarer malignant nodules that need immediate treatment to achieve survival benefits.

Increased attention has also been given to the use of magnetic resonance imaging (MRI) in the detection and screening of malignant nodules in recent years [7,8,9]. Turbo spin echo (SE)-based and gradient echo (GRE)-based techniques are important MRI sequences that are widely used to detect pulmonary nodules [10]. Motion artifacts due to respiration and heartbeat pose a particular challenge. The problem is currently addressed using a respiratory-navigated sequence with radial k-space acquisition, commonly known as MultiVane (Philips, Best, The Netherlands), fBLADE (Siemens, Erlangen, Germany), or PROPELLER (GE, Milwaukee, WI, USA), which provides excellent T2 contrast as it is insensitive to cardiac motion [11]. Besides the obvious advantage of radiation absence, studies concluded that MRI has similar sensitivity for nodule detection, with sensitivity and specificity even higher in malignant than in benign lesions compared to CT [12,13,14,15]. However, it remains a question of whether an MRI of the lungs could be used as a stand-alone diagnostic tool or in combination with CT to classify pulmonary nodules.

Artificial intelligence has a long history in the field of pulmonary nodule detection and classification dating back to the 1960s [16,17]. In 2012, Lambin and colleagues coined the term radiomics to describe quantitative imaging feature extraction to achieve better diagnostic performance [18]. Pathological studies have demonstrated increased heterogeneity within malignant pulmonary nodules, which are not visible to the naked eye on radiological examination but can be quantified with radiomics [19,20,21]. Therefore, the purpose of this study was to compare the performance of radiomic features analysis and radiologists in classifying pulmonary nodules based on stand-alone and complementary CT and MRI imaging.

## 2. Materials and Methods

### 2.1. Study Design and Sample

**Figure 1:**
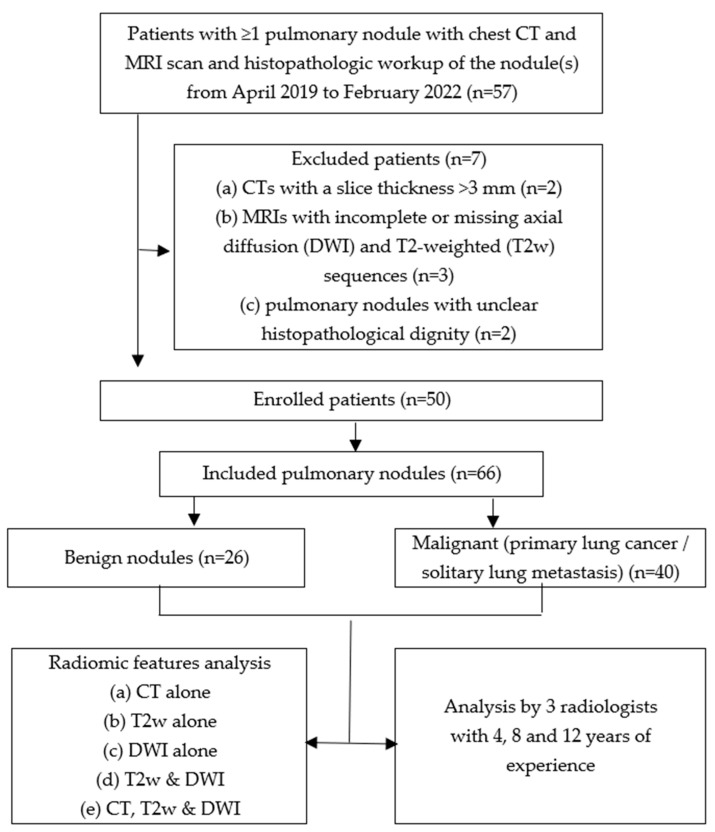
Inclusion and exclusion criteria for the patient cohort.

The study was approved by the medical–ethical committee, and informed consent was waived because of the retrospective collection of study data (RWTH Aachen University Hospital, Aachen, Germany). The study was performed in accordance with relevant guidelines and regulations and contemporary data protection laws. The entire cohort dataset was acquired from April 2019 to February 2022, including institutional picture archiving and communication system records (IntelliSpace PACS; Philips, Best, The Netherlands), using a standardized query for patients with pulmonary nodules who underwent standard-dose contrast-enhanced or non-enhanced chest CT scans and non-enhanced lung MRI. A radiologist with 5 years of experience in thoracal imaging screened patients with at least one pulmonary nodule who had undergone surgical resection and histopathologic examination of the lesion to determine benignity/malignancy. No distinction was made between primary or secondary lung malignancy. Exclusion criteria were as follows: (a) CTs with a slice thickness of >3 mm; (b) MRIs with incomplete or missing axial diffusion-(DWI) and T2-weighted (T2w) sequences; (c) pulmonary nodules with unclear histopathological results. If multiple pulmonary nodes were detected on CT, only histopathologic examined nodules were included in the study. For each patient, characteristics such as age, sex, average diameter, and pulmonary lobe were determined. Figure 1 provides an overview of the inclusion and exclusion criteria.

### 2.2. CT Parameters

All chest CTs were performed with 128-row spiral CT scanners (Somatom x.Site or Somatom Force, Siemens Medical Systems, Erlangen, Germany). The scans were acquired in a craniocaudal direction during a single-breath-hold either contrast-enhanced or non-enhanced. If contrast-enhanced CT was performed, a 1.0 mL/kg body weight bolus of iopromide 370 mg/mL (Ultravist, Bayer, Leverkusen, Germany) was injected intravenously by a power injector with an acquisition time of 75 s. Table 1 shows further technical details.

### 2.3. MRI Parameters

All chest MRIs were performed according to a standardized protocol using a 1.5 Tesla MRI system (Ambition or Ingenia, Philips, Best, The Netherlands) with a 32-element body surface coil. The standardized protocol contained axial and coronal T2w MultiVane-XD (MVXD), axial diffusion-weighted spin echo (SE), and axial T2w turbo spin echo (TSE) with and without fat saturation. Table 1 describes the detailed parameters of the pulse sequences used for further analysis in this study.

### 2.4. Image Segmentation

Two-dimensional manual segmentation in axial orientation was performed by a radiologist with 5 years of experience in thoracal imaging, using ITK-SNAP 3.6.0 (www.itksnap.org accessed on 26 July 2021) [22]. The segmentations delineated the visible borders of each pulmonary nodule in the pulmonary window of CT images, in T2w MVXD sequences, and in DWI SE sequences at a b-value of 800 s/mm^2^. In DWI SE sequences, a second segmentation of similar size was drawn in the dorsal subcutaneous fat tissue for harmonization purposes. All segmentations were validated by a senior radiologist with 12 years of experience in thoracal CTs.

### 2.5. Radiomic Features

Radiomic features were extracted using a PyRadiomics 3.0.1 framework [23] and based on feature definitions described by the Imaging Biomarker Standardization Initiative (IBSI) [24]. They included first-order statistical features, shape-based features, and texture features (gray level co-occurrence matrix, gray level run length matrix, gray level size zone matrix, neighboring gray-tone difference matrix, and gray level dependence matrix). A total of 105 radiomic features were extracted from each segmentation.

### 2.6. Development of a Prediction Model

Firstly, the radiomic features of CT imaging were evaluated for their value by differentiating pulmonary nodules into benign or malignant. To reduce the number of features, backward selection was employed, i.e., the prediction model was tasked to reduce the 105 features one by one, iteratively eliminating features with the lowest discriminatory value until only six features remained. To account for data scarcity, patient-by-patient leave-one-out cross-validation (LOOCV) was used. That is, the prediction model was trained repeatedly by leaving out one patient from the training set and training with the remaining set of patients until an independent prediction could be obtained for each patient. The backward selection process was then repeated for radiomic features in T2w MVXD sequences and again for DWI SE sequences at a b-value of 800 s/mm^2^. The three features with the highest discriminatory value resulting from separate training with T2w MVXD and DWI SE segmentations were then combined. The total accuracy of the six features was evaluated in a test set consisting of images corresponding to T2w and DWI MRI scans. Lastly, the two features with the highest discrimination value resulting from separate training with segmentations from T2w MVXD, DWI SE, and CT imaging were combined, and the total accuracy of the six features was evaluated in a test set consisting of images corresponding to CT, T2w, and DWI scans.

### 2.7. Human Reader Analysis

For comparative purposes, the same 2D image slices used for radiomic feature extraction were presented to three radiologists with 4, 8, and 12 years of experience in evaluating pulmonary nodules. Consistent with the radiomic analysis, the radiologists were presented with the standalone CT, T2w, and DWI datasets, followed by image sets corresponding to T2w and DWI as well as CT, T2w, and DWI, each in random order. They were requested to classify each nodule as benign or malignant. In the event of an interrater discrepancy, the majority vote was recorded.

### 2.8. Statistical Analysis

All the statistical analyses were performed using the Python packages SciPy 1.7.0 [25] and NumPy 1.21.0 [26]. Confusion matrices were calculated for each model. The performance of radiomics and human readers was compared by calculating accuracy, sensitivity, specificity, positive predictive value, and negative predictive value with Clopper–Pearson confidence intervals.

## 3. Results

In this study, 57 patients with ≥1 pulmonary nodule, chest CT and MRI scans, and a histopathologic workup of the nodule were screened for eligibility. The final group comprised 50 patients with a mean age of 63 years with a standard deviation of 10 years. Female patients totaled 18/50 (36%). Within this group, 66 pulmonary nodules were found with an average diameter of 0.9 cm. There was a slightly higher incidence in the right lung with 17 (26%) in the right upper lobe, 7 (11%) in the right middle lobe, and 12 (18%) in the right lower lobe compared to 18 (27%) in the left upper lobe and 12 (18%) in the left lower lobe. Of the 66 pulmonary nodules, 26 (39%) were benign and 40 (61%) were malignant, including 16 (24%) primary lung carcinomas and 24 (36%) solitary lung metastases. Table 2 summarizes the epidemiologic, clinical, and histological characteristics.

All 66 pulmonary nodules were visible to radiologists on CT. By contrast, only 61 (92.4%) of the nodules were visible in the T2w-weighted axial MultiVane XD sequence, and the five nodules that were not detectable turned out to be benign. Only 42 (63.6%) of the nodules were recovered in the axial diffusion-weighted spin echo sequence, with nodule detection in DWI associated with an increased likelihood of malignancy. The detection rate is summarized in Table 3.

**Figure 2:**
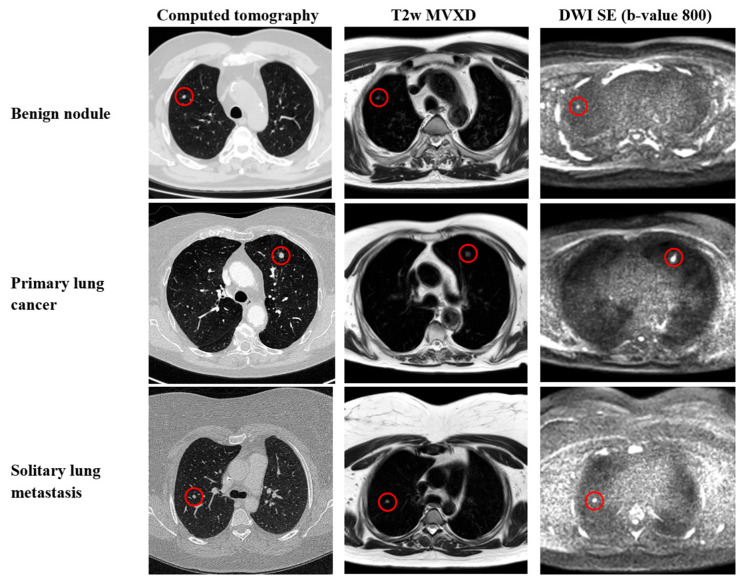
Examples of benign and malignant pulmonary nodules indicated by red circles in different image datasets. The benign nodule is a hamartoma located in the right upper lobe. Primary lung cancer is a moderately differentiated squamous cell carcinoma in the left upper lobe. Solitary lung metastasis is from synovial sarcoma in the right upper lobe.

Figure 2 shows illustrative examples of CT images and T2-weighted and DWI MRI sequences of a benign nodule, primary lung cancer, and solitary lung metastasis.

The backward selection process redacted the initial 105 radiomic features down to six features with the highest discriminatory values for benign and malignant in each image dataset (CT, T2w sequence, and DWI sequence), as shown in Table 4. For the combined analysis of T2w and DWI datasets, the top three radiomic features from the T2w and DWI datasets alone were used. For the combined analysis of CT, T2w, and DWI datasets, the top two radiomic features from the datasets of CT alone, T2w alone, and DWI alone were used, respectively.

A description of radiomic features with the highest discriminatory values found in each dataset is shown in Table 5.

The radiomic features analysis of the separate modalities and sequences showed the highest accuracy for the CT dataset (ACC 0.68; 95% CI: 0.56, 0.79), followed by the T2w (ACC 0.65; 95% CI: 0.52, 0.77) and DWI datasets (ACC 0.61; 95% CI: 0.48, 0.72). Human readers achieved the highest accuracy based on DWI (ACC 0.73; 95% CI: 0.60, 0.83), followed by T2w (ACC 0.68; 95% CI: 0.54, 0.78) and CT (ACC 0.59; 95% CI: 0.46, 0.71). When the T2w and DWI datasets were available for combined radiomic features analysis, the accuracy increased slightly (ACC 0.73; 95% CI: 0.60, 0.83) and when CT and MRI image data were included, the accuracy of radiomic features analysis further increased (ACC 0.83; 95% CI: 0.72, 0.91). By contrast, radiologists’ accuracy remained essentially the same for the combined image information of T2w and DWI (ACC 0.70; 95% CI: 0.57, 0.80) compared to T2w or DWI alone. Given the combined image information from CT, T2W, and DWI, radiologists’ accuracy displayed no improvement (ACC 0.64; 95% CI: 0.51–0.75). Table 6 provides a detailed comparison between radiomic analysis and radiologists’ results.

## 4. Discussion

Differentiating malignant from benign pulmonary nodules is a common diagnostic challenge for radiologists. Recent advances in MRI for evaluating pulmonary nodules have been increasingly used as a complementary or even stand-alone imaging modality to computed tomography. At the same time, machine-learning tools have also been presented as supporting tools for radiologists. Therefore, our objective was to compare the performance of human readers to radiomic feature analysis based on stand-alone and complementary CT and MRI imaging in classifying pulmonary nodules.

The study results show that the accuracy of radiomic feature analysis can increase if a combination of CT, T2w, and DWI is used (ACC 0.83) instead of CT (ACC 0.68), T2w (ACC 0.65), or DWI (ACC 0.61) alone. Interestingly, combining CT and MRI image datasets (ACC 0.64) did not significantly improve accuracy in human readers compared to CT (ACC 0.59), T2w (ACC 0.68), or DWI (ACC 0.73) alone. Each dataset consisted of images depicting the same 66 lung lesions of which 26 were benign and 40 were malignant (16 primary lung cancer and 24 solitary lung metastases). The mean diameter of the pulmonary nodules was 0.9 cm (SD 0.4 cm), and the patients were 65 years old (SD 10 years) on average. Based on these results, the supportive use of radiomic analysis in multimodal CT and MRI assessment of pulmonary nodules should be considered and further investigated to potentially improve radiologists’ assessments and decrease unnecessary biopsy rates or resections.

Many studies have already demonstrated the value of analyzing radiomic features in CT and MRI datasets in evaluating pulmonary nodules [27,28,29,30,31,32]. This study is the first to show that by combining quantitative image information from CT, T2w, and DWI datasets in a backward selection process, assessing pulmonary nodules through radiomics analysis is superior to that of one modality alone, even exceeding human readers’ performance.

A decisive factor for the comparatively low performance of human readers in this study is probably the small size of the nodules, with a mean of 0.9 cm. Since small nodules generally have a regular and compact shape, shape features that are easier for the human eye to recognize play a subordinate role, whereas texture features are of greater importance [33]. These observations are also reflected in the final six feature radiomics set found in this study. In this set, there are four first-order features concerning intensities and texture, one GLCM feature, one GLRLM feature, and not a single shape feature. Therefore, the transferability of the study results to larger nodules is limited, and results should be viewed in the context of small pulmonary nodules.

Another important limitation of this study is that we used data from only one institution. In future studies, the true predictive power of the current method should be assessed with an independent test dataset. Further limitations include the retrospective nature of the study and the small dataset of pulmonary nodules.

## 5. Conclusions

Quantitative image information from axial CT datasets or the DWI- or T2-weighted MRI datasets alone allows the assessment of pulmonary nodules by radiomics analysis compared to human readers’ performance. By providing image information from CT and MRI sequences, radiomics analysis is better than using a single modality and even surpasses the performance of human readers. Therefore, complementary CT and MRI assessment by radiomic features analysis can potentially reduce unnecessary biopsies or resections.

## Figures and Tables

**Table 1:** Technical data CT and MRI protocol.

Type of CT Scanner	Somatom x.Site or Somatom Force, Siemens Medical System, Erlangen, Germany	
Orientation	axial	
Direction	craniocaudal	
Section thickness	1/0.7 mm or 3/2 mm	
Tube voltage	120 kV	
Pitch factor	0.6	
Section collimation	128 mm	
**Type of MRI Scanner**	**1.5 T Ambition/Ingenia; Philipps Healthcare, Best, The Netherlands**	
Pulse sequence type	2D T2w MVXD	2D DWI SE
Orientation	axial	axial
Acquisition matrix	448 × 78	160 × 155
Field of view	360 mm	380 mm
Section thickness	5 mm	5 mm
TR	2500	2463.1
TE	110	86.7
b-value	0	50, 400, 800
Breath compensation	Respiratory triggering	Respiratory triggering

**Table 2:** Epidemiologic, clinical, and histological characteristics.

Cohort size, no.	50
Age (years), mean (SD)	63 (10)
Gender, no. (%)	
Male	32 (64)
Female	18 (36)
Pulmonary nodules, no.	66
Diameter (cm), mean (SD)	0.9 (0.4)
Location, no. (%)	
Right upper lobe	17 (26)
Right middle lobe	7 (11)
Right lower lobe	12 (18)
Left upper lobe	18 (27)
Left lower lobe	12 (18)
Histological typing, no. (%)	
Benign nodule	26 (39)
Primary lung cancer	16 (24)
Solitary lung metastasis	24 (36)

**Table 3:** Detectability of pulmonary nodules in given image datasets by human investigators.

Computed tomography (CT), no. (%)	66/66 (100)
T2w MVXD (T2W), no. (%)	61/66 (92.4)
Benign nodule	21/26 (80.8)
Malign (primary lung cancer or solitary lung metastasis)	40/40 (100)
DWI SE (b-value 800 s/mm^2^), no. (%)	42/66 (63.6)
Benign nodule	7/26 (26.9)
Malign (primary lung cancer or solitary lung metastasis)	35/40 (87.5)

**Table 4:** Top six radiomic features ranked by observed importance in single-image datasets.

	CT Alone	T2w Alone	DWI Alone	
**CT, T2w & DWI**	1. Difference Variance	1. 10th percentile	1. Mean	**T2w and DWI**
	2. Correlation	2. Skewness	2. Long Run Low Gray Level Emphasis	
	3. Coarseness	3. Dependence Entropy	3. Gray Level Non Uniformity	
	4. Complexity	4. Total Energy	4. Small Area Emphasis	
	5. Zone Entropy	5. Interquartile Range	5. Dependence Non Uniformity Normalized	
	6. 90th percentile	6. Dependence Non Uniformity Normalized	6. Large Dependence High Gray Level Emphasis	

**Table 5:** Description of radiomic features with the highest discriminatory values in this study [24].

**Difference Variance**	First-order feature measuring heterogeneity by placing a higher weight on differing intensity level pairs that deviate more from the mean.
**Correlation**	Gray level co-occurrence matrix (GLCM) feature measuring the linear dependency of gray level values on their respective voxels in the GLCM.
**Coarseness**	Neighboring gray tone difference matrix (NGTDM) feature measuring the average difference between the center voxel and its neighborhood.
**Complexity**	NGTDM feature measuring the number of primitive components in the image.
**Zone Entropy**	GLCM feature measuring the uncertainty/randomness in the distribution of zone sizes and gray levels.
**90th percentile**	First-order feature measuring the 90th percentile of voxel intensities within the image region.
**10th percentile**	First-order feature measuring the 10th percentile of voxel intensities within the image region.
**Skewness**	First-order feature measuring the asymmetry of the value distribution about the mean value.
**Dependence Entropy**	Gray level dependence matrix (GLDM) feature measuring the randomness/variability of gray level dependencies in an image.
**Total Energy**	First-order feature measuring the magnitude of voxel values scaled by the volume of the voxel.
**Interquartile Range**	First-order feature measuring the difference between the 75th and 25th percentile of the image array.
**Dependence Non Uniformity Normalized**	GLDM feature measuring the similarity of dependence throughout the image, with a lower value indicating more homogeneity among dependencies in the image.
**Mean**	First-order feature measuring the average gray level intensity within the ROI.
**Long Run Low Gray Level Emphasis**	Gray level run length matrix (GLRLM) feature measuring the joint distribution of long-run lengths with lower gray-level values.
**Gray Level Non Uniformity**	GLRLM feature measuring the similarity of gray-level intensity values in the image.
**Small Area Emphasis**	Gray level size zone matrix (GLSZM) feature measuring the distribution of small size zones, with a greater value indicative of smaller size zones and more fine textures.
**Large Dependence High Gray Level Emphasis**	GLDM feature measuring the joint distribution of large dependencies with higher gray-level values.

**Table 6:** Comparison between radiomic features analysis and radiologists’ results based on the image data provided. The 95% confidence interval is shown in brackets.

		CT Alone	T2w Alone	DWI Alone	T2w/DWI	CT/T2w/DWI
**Radiomic analysis**	Sensitivity	0.68 (0.51–0.81)	0.85 (0.70–0.94)	0.43 (0.27–0.59)	0.68 (0.51–0.81)	0.95 (0.83–0.99)
	Specificity	0.69 (0.48–0.86)	0.35 (0.17–0.56)	0.89 (0.70–0.98)	0.81 (0.61–0.94)	0.65 (0.44–0.83)
	Positive Predictive Value	0.77 (0.60–0.90)	0.67 (0.52–0.79)	0.85 (0.62–0.97)	0.84 (0.67–0.95)	0.81 (0.67–0.91)
	Negative Predictive Value	0.58 (0.39–0.76)	0.60 (0.32–0.84)	0.50 (0.35–0.65)	0.62 (0.44–0.78)	0.90 (0.67–0.99)
	Accuracy	0.68 (0.56–0.79)	0.65 (0.52–0.77)	0.61 (0.48–0.72)	0.73 (0.60–0.83)	0.83 (0.72–0.91)
**Radiologists**	Sensitivity	0.75 (0.59–0.87)	0.70 (0.55–0.83)	0.73 (0.56–0.85)	0.83 (0.67–0.93)	0.80 (0.64–0.91)
	Specificity	0.35 (0.17–0.56)	0.62 (0.41–0.80)	0.73 (0.52–0.88)	0.50 (0.30–0.70)	0.39 (0.20–0.59)
	Positive Predictive Value	0.64 (0.49–0.77)	0.74 (0.57–0.87)	0.81 (0.64–0.92)	0.72 (0.57–0.84)	0.67 (0.52–0.80)
	Negative Predictive Value	0.47 (0.25–0.71)	0.57 (0.37–0.76)	0.63 (0.44–0.80)	0.65 (0.41–0.85)	0.56 (0.31–0.79)
	Accuracy	0.59 (0.46–0.71)	0.68 (0.54–0.78)	0.73 (0.60–0.83)	0.70 (0.57–0.80)	0.64 (0.51–0.75)

## Data Availability

The data presented in this study are available upon request from the corresponding author.
